# TACE–HAIC combined with apatinib and camrelizumab for unresectable hepatocellular carcinoma with portal vein tumor thrombus: a multicenter retrospective study

**DOI:** 10.3389/fimmu.2026.1917667

**Published:** 2026-10-01

**Authors:** Xuexian Zhang, Shangbo Shi, Yang Su, Yuanjin Ma, Qikun Guo, Xuerou Meng, Ruidong Wang, Jian Long, Jidong Yang, Bin Xiong

**Affiliations:** 1 Department of Interventional Medicine, The First Affiliated Hospital of Guangzhou Medical University, Guangzhou, Guangdong, China; 2 Department of Interventional Medicine, The Second Affiliated Hospital of Wenzhou Medical University, Wenzhou, Zhejiang, China; 3 Hepatobiliary and Pancreatic Interventional Treatment Center, Division of Hepatobiliary and Pancreatic Surgery, The First Affiliated Hospital, Zhejiang University School of Medicine, Hangzhou, Zhejiang, China; 4 Department of Interventional Radiology, The First Affiliated Hospital of Kunming Medical University, Kunming, Yunnan, China; 5 Department of Interventional Radiology, Qujing Second People’s Hospital, Qujing, Yunnan, China; 6 Department of Vascular and Interventional Radiology, Jingmen People’s Hospital, Jingchu University of Technology Affiliated Jingmen People’s Hospital, Jingmen, Hubei, China

**Keywords:** apatinib, camrelizumab, hepatic arterial infusion chemotherapy, portal vein tumor thrombus, transarterial chemoembolization, unresectable hepatocellular carcinoma

## Abstract

**Background and objectives:**

Unresectable hepatocellular carcinoma (uHCC) with portal vein tumor thrombus (PVTT) indicates a poor prognosis. This study aims to evaluate the efficacy and safety of transarterial chemoembolization (TACE) combined with hepatic arterial infusion chemotherapy (HAIC), apatinib, and camrelizumab in patients with uHCC accompanied by PVTT.

**Methods:**

This multicenter retrospective study included patients with uHCC and PVTT who were treated at five tertiary medical centers in China between December 2021 and April 2025. Propensity score matching (PSM) was performed to minimize potential confounding bias. Treatment efficacy was assessed by objective response rate (ORR), disease control rate (DCR), progression-free survival (PFS), and overall survival (OS). The primary endpoints were PFS and OS, whereas secondary endpoints included ORR and safety.

**Results:**

Among the 222 patients who met the eligibility criteria, 105 received TACE-HAIC combined with apatinib and camrelizumab (THAC group), and 117 received TACE combined with apatinib and camrelizumab (TAC group). After PSM, 86 matched patients were included in each group, and baseline characteristics were well balanced between the two groups. The THAC group achieved significantly higher intrahepatic tumor ORR (70.9% vs. 47.7%, P = 0.002) and DCR (94.2% vs. 79.1%, P = 0.004) than the TAC group. Similarly, for PVTT, the THAC group demonstrated significantly higher ORR (61.6% vs. 41.9%, P = 0.009) and DCR (90.7% vs. 74.4%, P = 0.005). The median PFS was significantly longer in the THAC group than in the TAC group (12 months vs. 7 months; HR = 0.287; 95%CI: 0.204–0.403; P<0.001). Likewise, the median OS was significantly prolonged in the THAC group (24 months vs. 16 months; HR = 0.198; 95%CI: 0.135–0.291; P<0.001). The incidence of adverse events did not differ significantly between the two groups (P>0.05). All adverse events were manageable, and no grade 5 adverse events were observed.

**Conclusions:**

In patients with uHCC accompanied by PVTT, the THAC regimen was associated with superior survival outcomes compared with the TAC regimen while maintaining an acceptable safety profile. These findings suggest that THAC may represent a feasible and effective therapeutic option for this patient population.

## Introduction

Hepatocellular carcinoma (HCC) is the sixth most commonly diagnosed cancer worldwide and the third leading cause of cancer-related death (1). Large (5.1–10 cm in diameter) and huge (>10 cm in diameter) HCCs are often considered unresectable because adequate surgical margins cannot be achieved and the estimated future liver remnant volume is less than 30% after resection (2).

HCC is characterized by a high propensity for vascular invasion, frequently involving the portal and hepatic veins and resulting in the formation of tumor thrombi, among which portal vein tumor thrombus (PVTT) is the most common (3). Approximately 44%–62% of patients with HCC develop PVTT, which is recognized as a major predictor of poor prognosis (4). The incidence of PVTT is particularly high in patients with large tumors (>5 cm in diameter), reaching 58.5% (5).

Current international guidelines consider HCC with PVTT to be associated with a low likelihood of cure and recommend systemic therapy as the primary treatment option. However, the therapeutic efficacy of systemic therapy alone remains limited, and optimizing multidisciplinary treatment strategies to improve survival outcomes continues to be a major clinical challenge (6, 7).

Several studies have demonstrated that transarterial chemoembolization (TACE) is an effective locoregional treatment for patients with HCC and PVTT (8). In addition, Chinese clinical practice guidelines recommend TACE as an alternative therapeutic option for this patient population (9).

Hepatic arterial infusion chemotherapy (HAIC) is a locoregional treatment modality that delivers chemotherapeutic agents directly to the tumor through an indwelling microcatheter, thereby increasing local drug concentrations while substantially reducing systemic toxicity (10). Given its ability to achieve sustained intratumoral drug exposure, HAIC has emerged as a promising therapeutic approach for patients with advanced HCC. Several studies have demonstrated its efficacy in patients with HCC accompanied by PVTT (11–13). Furthermore, the Japan Society of Hepatology (JSH) guidelines explicitly recommend HAIC as one of the first-line treatment options for patients with portal vein involvement (14).

Apatinib is a small-molecule tyrosine kinase inhibitor (TKI) that suppresses tumor angiogenesis and inhibits tumor growth by selectively targeting vascular endothelial growth factor receptor 2 (VEGFR-2) (15). The AHELP study demonstrated that apatinib monotherapy as a second-line or later treatment for advanced hepatocellular carcinoma significantly improved survival outcomes and achieved a considerable objective response rate (16).

Camrelizumab is a programmed cell death protein 1 (PD-1) inhibitor that has been approved by the Chinese National Medical Products Administration for the treatment of HCC (17). The IMbrave150 trial was the first to demonstrate that the combination of the anti–programmed death ligand 1 (PD-L1) antibody atezolizumab and the anti–vascular endothelial growth factor (VEGF) antibody bevacizumab provided a significant survival benefit over sorafenib in patients with unresectable HCC, establishing immune checkpoint blockade combined with antiangiogenic therapy as a landmark treatment strategy for HCC (18).

Subsequently, accumulating evidence has further supported the clinical value of combining targeted therapy with immunotherapy in patients with unresectable HCC. Apatinib plus camrelizumab has demonstrated favorable antitumor activity and survival benefits in this population (19–21). Based on these findings, the combination has been approved in China as a first-line treatment for unresectable HCC (22).

However, to the best of our knowledge, clinical evidence regarding the use of TACE-HAIC combined with apatinib and camrelizumab in patients with unresectable hepatocellular carcinoma (uHCC) and portal vein tumor thrombus (PVTT) remains limited. Therefore, we conducted this multicenter retrospective study to assess the efficacy and safety of this combination regimen in this patient population.

## Materials and methods

### Study design and patient selection

We retrospectively analyzed the clinical data of patients with unresectable hepatocellular carcinoma (uHCC) accompanied by portal vein tumor thrombus (PVTT) who received either TACE-HAIC combined with apatinib and camrelizumab or TACE combined with apatinib and camrelizumab at five tertiary medical centers in China between December 2021 and April 2025.

The inclusion criteria were as follows: (1) age 18–80 years; (2) diagnosis of HCC according to the criteria of the European Association for the Study of the Liver (EASL) guidelines or histopathological confirmation by liver biopsy; (3) PVTT confirmed by computed tomography (CT) or magnetic resonance imaging (MRI); (4) Child–Pugh class A or B liver function; and (5) an Eastern Cooperative Oncology Group performance status (ECOG PS) score of 0–1.The exclusion criteria were as follows: (1) concomitant malignancies other than HCC; (2) severe underlying cardiac, pulmonary, or renal disease; (3) receipt of any prior antitumor treatment before the initial TACE procedure, including surgery, radiotherapy, chemotherapy, or immunotherapy; and (4) incomplete clinical follow-up data. Patients were divided into two groups according to whether they received HAIC: the TACE-HAIC combined with apatinib and camrelizumab group (THAC group) and the TACE combined with apatinib and camrelizumab group (TAC group).

PVTT was classified according to the Vp classification system proposed by the Liver Cancer Study Group of Japan (LCSGJ) (23). Vp1 was defined as PVTT confined to the portal vein distal to the second-order branch; Vp2 as PVTT involving the second-order branch of the portal vein; Vp3 as PVTT involving the first-order branch of the portal vein; and Vp4 as PVTT involving the main portal vein or the contralateral first-order branch.

This multicenter retrospective study was approved by the Institutional Review Board of each participating center and was conducted in accordance with the ethical principles of the 1975 Declaration of Helsinki. Owing to the retrospective nature of the study, the requirement for informed consent was waived by the ethics committees.

Treatment decisions were made on the basis of individual patient characteristics and physician discretion. At each participating center, treatment strategies were determined by a multidisciplinary team in accordance with the Barcelona Clinic Liver Cancer (BCLC) guidelines or the Chinese national guidelines for the diagnosis and treatment of liver cancer. Before the final treatment decision was made, patients and their families were informed of the advantages and disadvantages of the available treatment options, including potential therapeutic outcomes, financial burden, and treatment-related complications.

### Treatment protocol

#### TACE procedure

TACE procedures were performed by interventional radiologists with at least 10 years of experience at each participating center. All procedures were conducted in accordance with standard TACE protocols, and embolization was implemented using an emulsion of lipiodol and epirubicin hydrochloride. After local anesthesia, the right femoral artery was punctured using the Seldinger technique, and a 5-F vascular sheath (Terumo, Japan) was inserted. Subsequently, a 5-F Yashiro catheter (Terumo, Japan) was advanced under digital subtraction angiography (DSA) guidance into the celiac trunk and common hepatic artery for angiographic assessment of tumor location, size, number, and vascular supply. If no definite tumor-feeding artery was identified or tumor staining was incomplete, additional angiography of the superior mesenteric artery, renal artery, left gastric artery, inferior phrenic artery, and other potential collateral vessels was performed to identify the tumor-feeding arteries. After confirmation of the tumor blood supply, a 2.7-F RAPIDTHRU microcatheter (Hengrui, Suzhou, China) was superselectively advanced into the tumor-feeding artery. An emulsion of lipiodol and epirubicin hydrochloride was then slowly infused, followed by embolization with gelatin sponge particles until the embolization endpoint was achieved, which was defined as near stasis of blood flow in the tumor-feeding artery. If arterioportal fistulas or arteriovenous fistulas were identified during angiography, the microcatheter was superselectively advanced to the fistula site, and polyvinyl alcohol (PVA) particles were used to occlude the fistula before chemoembolization was performed.

#### HAIC procedure

In the THAC group, following completion of the TACE procedure, the microcatheter was retained in the proper hepatic artery, left hepatic artery, or right hepatic artery. After returning to the ward, patients received continuous HAIC through the indwelling microcatheter. HAIC was administered using a modified FOLFOX regimen (24). Specifically, oxaliplatin (85 mg/m²) was infused intra-arterially over 2–3 hours, followed by calcium folinate (400 mg/m²) administered intravenously over 1 hour and fluorouracil (400 mg/m²) delivered via intra-arterial bolus injection. Subsequently, fluorouracil was continuously infused intra-arterially at a dose of 2400 mg/m² over 46 hours. During follow-up, contrast-enhanced computed tomography (CT) or magnetic resonance imaging (MRI) was performed to assess tumor response. If viable tumor tissue or intrahepatic recurrence was detected, subsequent TACE or TACE-HAIC sessions were performed as needed according to the patient’s liver function status.

### Systemic therapy

Systemic therapy was initiated within 3 days after the first TACE procedure. Apatinib was administered orally at a dose of 250 mg once daily. Camrelizumab was administered intravenously at a dose of 200 mg for patients weighing >50 kg or 3 mg/kg for patients weighing ≤50 kg, every 2 weeks. In the event of treatment-related adverse events, dose reduction, temporary interruption, or permanent discontinuation of the study drugs was permitted according to the severity of the adverse events. For grade 3 or higher adverse events, dose reduction or temporary interruption of apatinib and temporary interruption of camrelizumab were allowed. Treatment could be resumed at the original dose after resolution of symptoms. If the adverse events remained intolerable despite these measures, the corresponding treatment was permanently discontinued.

### Follow-up

Standardized patient assessments were performed before each treatment session and at each routine follow-up visit. Patients were followed up monthly. Follow-up evaluations included contrast-enhanced liver CT or MRI, electrocardiography, laboratory examinations (including blood biochemistry, complete blood count, coagulation function, liver function, and serum alpha-fetoprotein [AFP] levels), as well as assessment of clinical symptoms and physical signs. The severity of adverse events was evaluated according to the National Cancer Institute Common Terminology Criteria for Adverse Events (CTCAE), version 5.0 (25). The final follow-up date was April 2026.

### Assessment of treatment efficacy

The primary endpoints of this study were progression-free survival (PFS) and overall survival (OS), whereas the secondary endpoints were objective response rate (ORR) and safety. PFS was defined as the time from the date of the first TACE procedure to the first occurrence of radiologically documented disease progression or death from any cause, whichever occurred first. Patients who had neither radiological disease progression nor death by the date of the last follow-up were censored at the date of their last available disease assessment. OS was defined as the interval from the date of the first TACE procedure to death from any cause or the last follow-up assessment. Intrahepatic tumor response and PVTT response were independently evaluated according to the modified Response Evaluation Criteria in Solid Tumors (mRECIST) by two radiologists at each center, each with more than 5 years of experience (26). In cases of disagreement, the more senior radiologist made the final determination. Tumor response was categorized as complete response (CR), partial response (PR), stable disease (SD), or progressive disease (PD). According to the mRECIST criteria, ORR was defined as the sum of CR and PR, whereas DCR was defined as the sum of CR, PR, and SD.

### Statistical analysis

Statistical analyses were performed using SPSS software (version 25.0; IBM Corp., Armonk, NY, USA) and R software (RStudio, version 4.2.2). Propensity score matching (PSM) was performed to reduce selection bias and balance baseline characteristics between the two groups. Propensity scores were estimated using a multivariable logistic regression model that included the following covariates: gender, age, HBV infection, Cirrhosis, Child Pugh class, AFP, Tumor diameter, Tumor number. PVTT grade, and Extrahepatic metastasis. A 1:1 nearest-neighbor matching algorithm without replacement was applied, with a caliper width of 0.02. Categorical variables were compared using the chi-square test or Fisher’s exact test, as appropriate. Survival outcomes were estimated using the Kaplan–Meier method and compared using the log-rank test. The median follow-up duration was estimated using the reverse Kaplan–Meier method. Survival curves were generated accordingly. Univariable and multivariable Cox proportional hazards regression analyses were performed to identify prognostic factors and conduct subgroup analyses. Covariates with a P value <0.1 in the univariable analysis were entered into the multivariable model. All statistical tests were two-sided, and a two-tailed P value <0.05 was considered statistically significant.

## Results

### Patient characteristics

After screening and eligibility assessment, a total of 222 patients were included in the study, comprising 105 patients in the THAC group and 117 patients in the TAC group. The patient selection process is shown in **Figure 1**. Following 1:1 propensity score matching (PSM), 86 patients remained in each group. Before matching, a significant difference in tumor number was observed between the two groups. After PSM, all baseline clinical characteristics were well balanced between the THAC and TAC groups, with no statistically significant differences observed (all P > 0.05). Detailed baseline characteristics before and after PSM are presented in **Table 1**. The probability-density plots (A) and Love plots (B) for covariate balance before and after PSM are shown in **Figure 2**.

**Figure 1:**
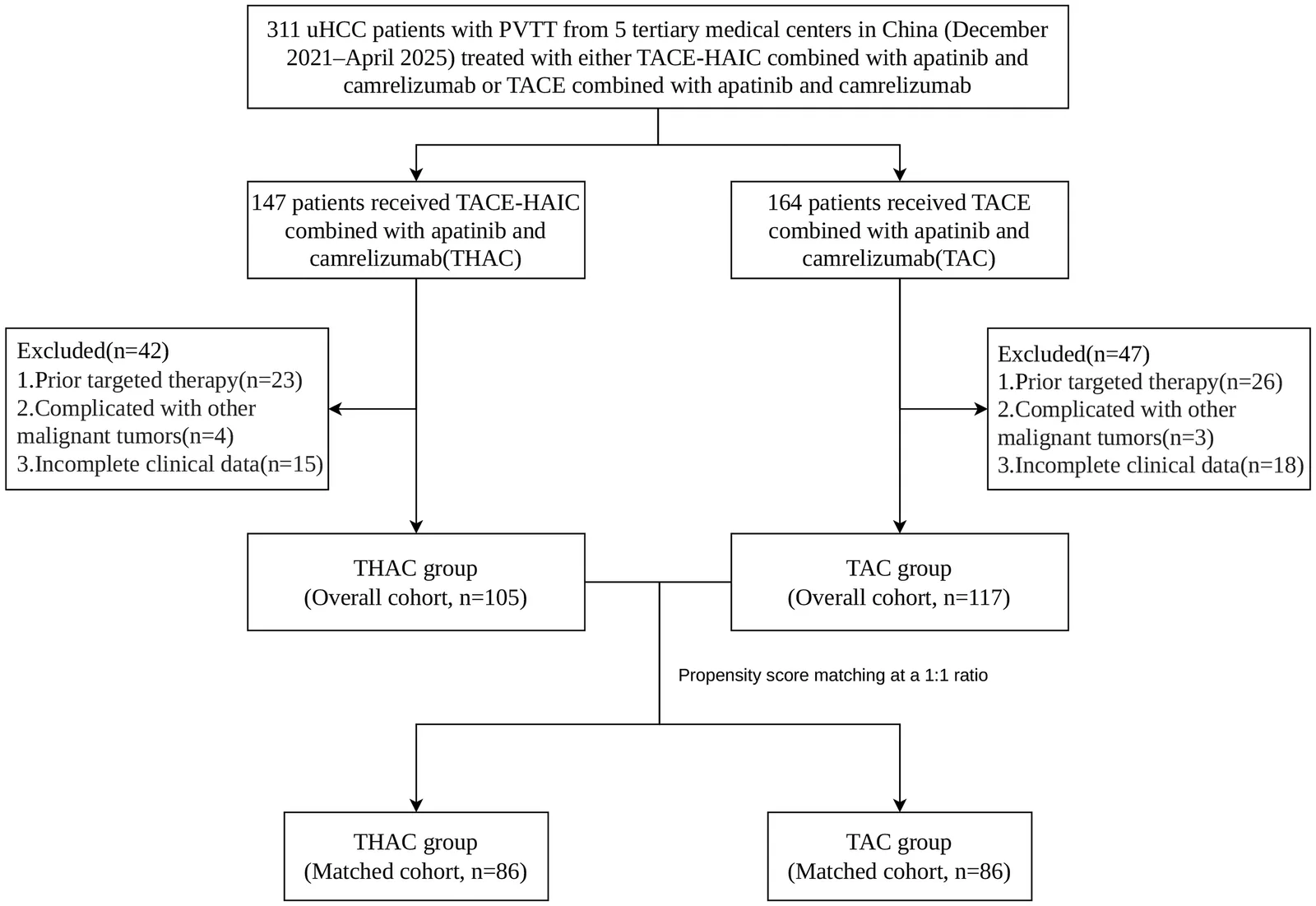
Flowchart of the patients selection process for this study.

**Table 1:** Comparison of baseline characteristics between the two groups.

Characteristics	Before PSM				After PSM			
	THAC group (n = 105)	TAC group (n = 117)	P-value	SMD	THAC group (n = 86)	TAC group (n = 86)	P-value	SMD
Gender			0.446	0.094			1.000	0.000
Male	97	111			81	81		
Female	8	6			5	5		
Age(y)			0.924	0.013			1.000	0.000
≤60	59	65			48	48		
>60	46	52			38	38		
HBV infection			0.741	0.043			1.000	0.000
Yes	89	101			75	75		
No	16	16			11	11		
Cirrhosis			0.688	0.053			0.843	0.030
Yes	83	95			70	71		
No	22	22			16	15		
Child-Pugh class			0.740	0.045			0.760	0.047
A	49	52			39	41		
B	56	65			47	45		
ECOG PS			0.547	0.081			1.000	0.000
0	50	51			40	40		
1	55	66			46	46		
AFP (ng/mL)			0.849	0.025			0.538	0.094
≤400	57	65			51	47		
>400	48	52			35	39		
Tumor diameter(cm)			0.701	0.051			0.392	0.135
5.1-10	72	83			65	60		
>10	33	34			21	26		
Tumor number			0.005	0.402			0.870	0.025
≤3	31	56			28	27		
>3	74	61			58	59		
PVTT grade			0.629	0.066			1.000	0.000
Vp1-2	24	30			21	21		
Vp3-4	81	87			65	65		
Extrahepatic metastasis			0.588	0.077			0.787	0.043
Yes	11	15			7	8		
No	94	102			79	78		

PSM, Propensity Score Matching; THAC, TACE-HAIC combined with apatinib plus camrelizumab therapy; TAC, TACE combined with apatinib plus camrelizumab therapy; HBV, Hepatitis B Virus; ECOG PS, Eastern Cooperative Oncology Group Performance Status; AFP, Alpha-fetoprotein; PVTT, Portal vein tumor thrombosis.

**Figure 2:**
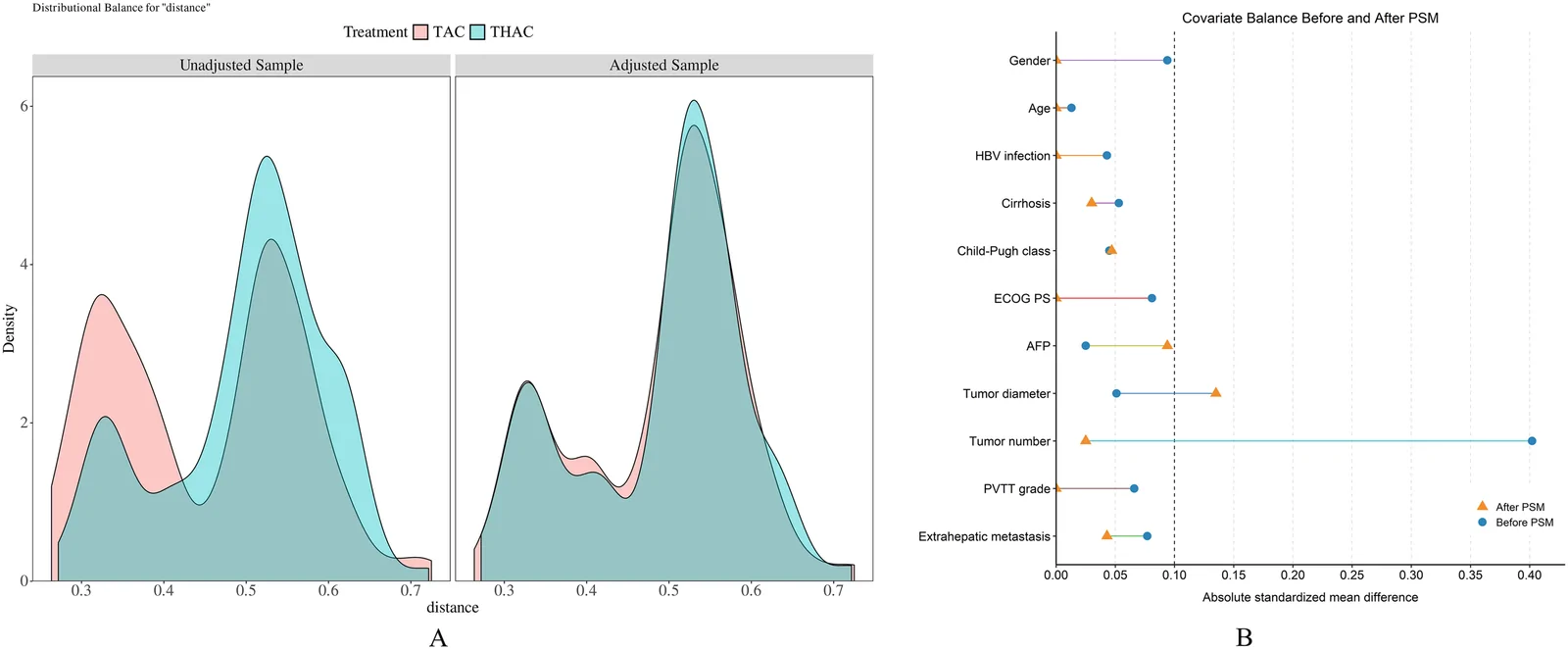
The probability-density plots **(A)** and Love plots **(B)** for covariate balance before and after PSM.

### Tumor responses

The best tumor responses of intrahepatic tumors and PVTT before and after PSM are summarized in **Table 2**. In the matched cohort, the THAC group achieved a significantly higher objective response rate (ORR) for intrahepatic tumors than the TAC group (70.9% vs. 47.7%, P = 0.002). Similarly, the disease control rate (DCR) was significantly higher in the THAC group than in the TAC group (94.2% vs. 79.1%, P = 0.004). With respect to PVTT, both the ORR (61.6% vs. 41.9%, P = 0.009) and DCR (90.7% vs. 74.4%, P = 0.005) were significantly higher in the THAC group than in the TAC group.

**Table 2:** Best tumor and PVTT response based on mRECIST between the two groups.

Tumor responses	Before PSM			After PSM		
	THAC group	TAC group	P-value	THAC group	TAC group	P-value
	(n = 105)	(n = 117)		(n = 86)	(n = 86)	
Intrahepatic tumor						
CR	18(17.1%)	9(7.7%)		13(15.1%)	7(8.1%)	
PR	57(54.3%)	45(38.5%)		48(55.8%)	34(39.5%)	
SD	22(21.0%)	40(34.2%)		20(23.3%)	29(33.7%)	
PD	8(7.6%)	23(19.6%)		5(5.8%)	16(18.6%)	
ORR(CR+PR)	75(71.4%)	54(46.2%)	<0.001	61(70.9%)	41(47.7%)	0.002
DCR(CR+PR+SD)	97(92.4%)	94(80.3%)	0.010	81(94.2%)	68(79.1%)	0.004
PVTT						
CR	10(9.5%)	5(4.3%)		7(8.1%)	3(3.5%)	
PR	56(53.4%)	43(36.8%)		46(53.5%)	33(38.4%)	
SD	29(27.6%)	41(35.0%)		25(29.1%)	28(32.6%)	
PD	10(9.5%)	28(23.9%)		8(9.3%)	22(25.5%)	
ORR(CR+PR)	66(62.9%)	48(41.0%)	0.001	53(61.6%)	36(41.9%)	0.009
DCR(CR+PR+SD)	95(90.5%)	89(76.1%)	0.004	78(90.7%)	64(74.4%)	0.005

PVTT, Portal vein tumor thrombosis; mRECIST, Modified response evaluation criteria in solid tumors; PSM, Propensity Score Matching; THAC, TACE-HAIC combined with apatinib plus camrelizumab therapy; TAC, TACE combined with apatinib plus camrelizumab therapy; CR, Complete response; PR, partial response; SD, Stable disease; PD, Progressive disease; ORR, Objective response rate; DCR, Disease control rate.

### Survival outcomes

Before PSM, the median follow-up duration was 34 months in the THAC group and was not reached in the TAC group according to the reverse Kaplan–Meier method. Survival analysis demonstrated that the THAC group achieved a significantly longer median progression-free survival (mPFS) than the TAC group (12 months vs. 8 months; HR = 0.270, 95% CI: 0.198–0.367; P<0.001) (**Figure 3A**). Similarly, the median overall survival (mOS) was significantly longer in the THAC group than in the TAC group (26 months vs. 17 months; HR = 0.151, 95% CI: 0.105–0.218; P<0.001) (**Figure 3B**). After PSM, the median follow-up duration was not reached in either the THAC or TAC group according to the reverse Kaplan–Meier method. The survival benefit of the THAC group remained significant. The median PFS was 12 months in the THAC group and 7 months in the TAC group (HR = 0.287, 95% CI: 0.204–0.403; P<0.001) (**Figure 3C**). Likewise, the median OS was significantly longer in the THAC group than in the TAC group (24 months vs. 16 months; HR = 0.198, 95% CI: 0.135–0.291; P<0.001) (**Figure 3D**).

**Figure 3:**
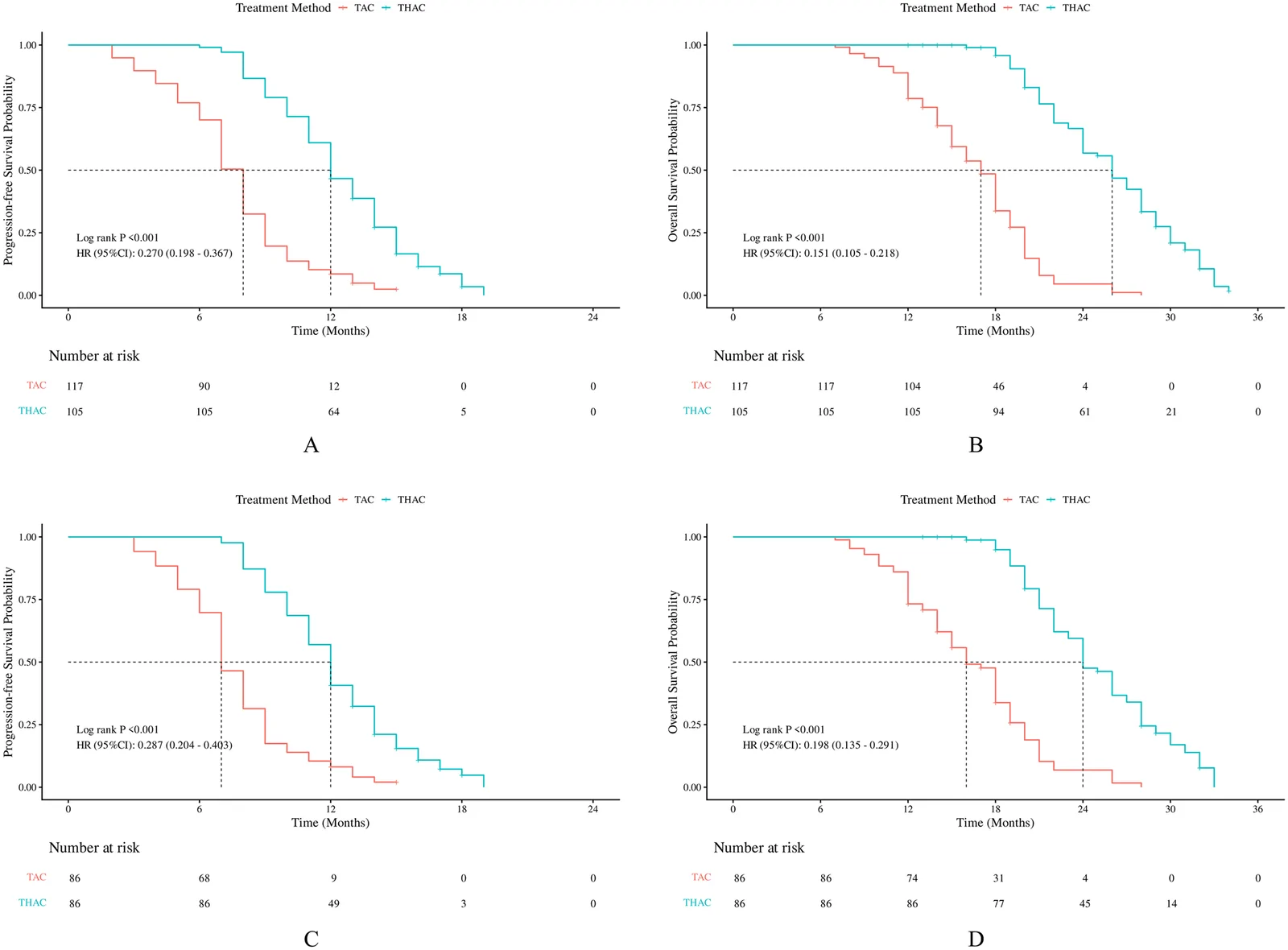
**(A)** The Kaplan_Meier curves comparing the progression_free survival between the THAC group and the TAC group without PSM_adjusted; **(B)** The Kaplan_Meier curves comparing the overall survival between the THAC group and the TAC group without PSM_adjusted; **(C)** Comparison of PSM_adjusted progression_free survival between the THAC group and TAC groups; **(D)** Comparison of PSM_adjusted overall survival between the THAC group and TAC groups.

Furthermore, additional survival analyses were performed according to the of PVTT grade, categorized as Vp1/2 and Vp3/4. Before PSM, patients with Vp1/2 PVTT achieved a significantly longer mPFS than those with Vp3/4 PVTT (11 months vs. 9 months; HR = 1.545, 95% CI: 1.107–2.156; P = 0.005) (**Figure 4A**). Similarly, the mOS showed a trend toward longer survival in patients with Vp1/2 PVTT compared with those with Vp3/4 PVTT (21 months vs. 20 months; HR = 1.393, 95% CI: 0.976–1.989; P = 0.050) (**Figure 4B**). After PSM, the survival difference according to PVTT grade remained significant. The mPFS was 11 months in the Vp1/2 group and 9 months in the Vp3/4 group (HR = 1.591, 95% CI: 1.088–2.327; P = 0.008) (**Figure 4C**). Likewise, the mOS was significantly longer in patients with Vp1/2 PVTT than in those with Vp3/4 PVTT (23 months vs. 20 months; HR = 1.753, 95% CI: 1.170–2.626; P = 0.003) (**Figure 4D**).

**Figure 4:**
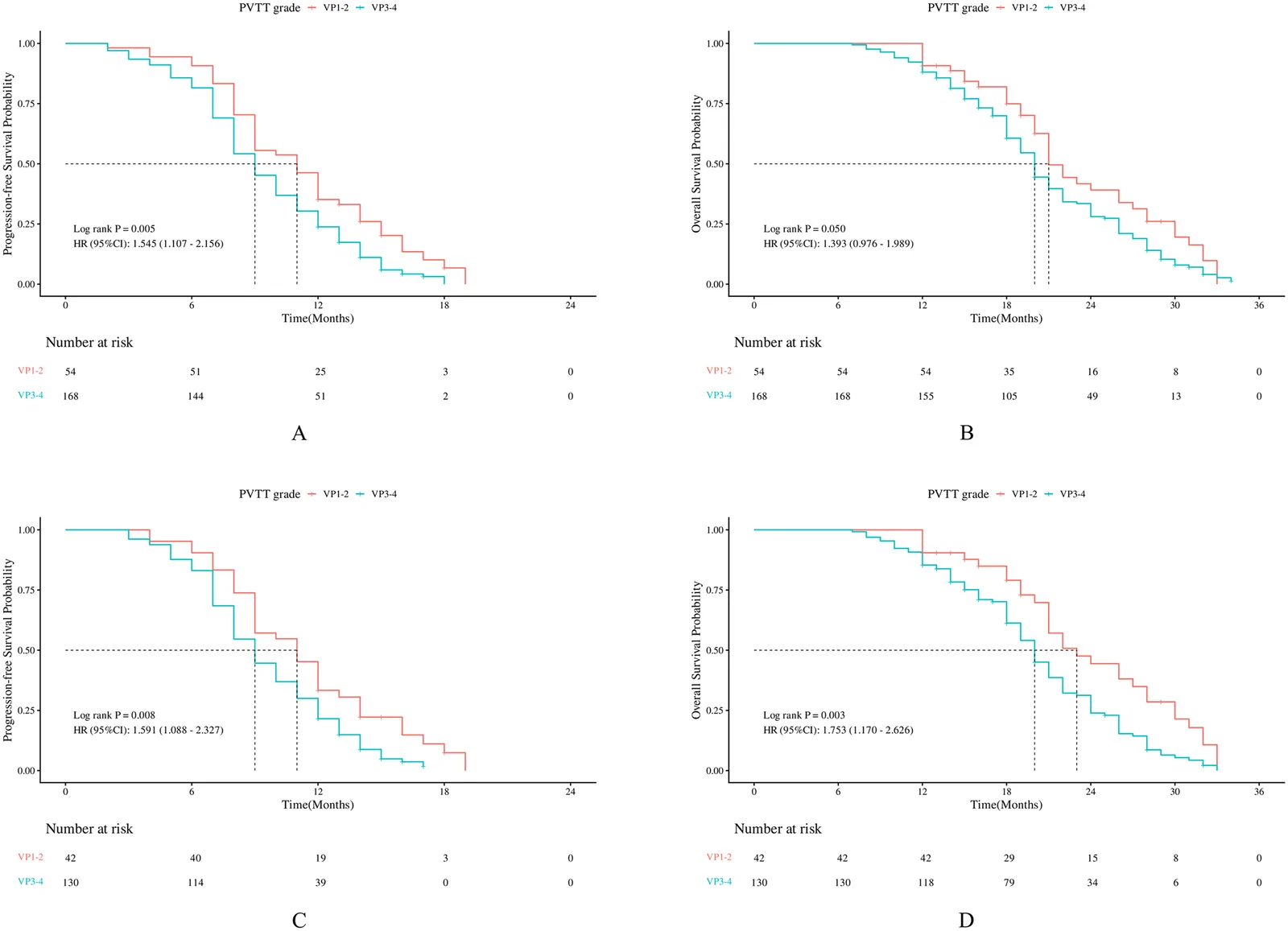
**(A)** The Kaplan_Meier curves comparing the progression_free survival between the Vp1_2 group and the Vp3_4 group without PSM_adjusted; **(B)** The Kaplan_Meier curves comparing the overall survival between the Vp1_2 group and the Vp3_4 group without PSM_adjusted; **(C)** Comparison of PSM_adjusted progression_free survival between the Vp1_2 group and Vp3_4 groups; **(D)** Comparison of PSM_adjusted overall survival between the Vp1_2 group and Vp3_4 groups.

### Predictors of PFS and OS

After PSM, multivariable Cox proportional hazards regression analysis identified tumor diameter (HR = 6.03, 95% CI: 3.56–10.20, P<0.001) and PVTT classification (HR = 2.01, 95% CI: 1.36–2.97, P<0.001) as independent risk factors for PFS, whereas treatment modality (HR = 0.20, 95% CI: 0.14–0.29, P<0.001) was identified as an independent protective factor. Detailed results are presented in **Table 3**. For OS, tumor diameter (HR = 4.01, 95% CI: 2.70–5.96, P<0.001), tumor number (HR = 1.49, 95% CI: 1.01–2.22, P = 0.046), PVTT classification (HR = 1.82, 95% CI: 1.19–2.78, P = 0.006), and extrahepatic metastasis (HR = 4.78, 95% CI: 2.65–8.62, P<0.001) were identified as independent risk factors, whereas treatment modality (HR = 0.13, 95% CI: 0.08–0.19, P<0.001) was identified as an independent protective factor. Detailed results are presented in **Table 4**.

**Table 3:** Predictors of progression-free survival after PSM.

Variables	Univariable analysis		Multivariable analysis	
	HR (95%CI)	*P*	HR (95%CI)	*P*
Treatment method				
TAC	1.00 (Reference)		1.00 (Reference)	
THAC	0.29 (0.20 ~ 0.40)	<0.001	0.20 (0.14 ~ 0.29)	<0.001
Gender				
Male	1.00 (Reference)			
Female	1.15 (0.58 ~ 2.26)	0.686		
Age(y)				
≤60	1.00 (Reference)			
>60	1.06 (0.77 ~ 1.45)	0.715		
HBV infection				
No	1.00 (Reference)			
Yes	0.76 (0.48 ~ 1.20)	0.236		
Cirrhosis				
No	1.00 (Reference)			
Yes	1.15 (0.76 ~ 1.75)	0.500		
Child-Pugh class				
A	1.00 (Reference)			
B	1.04 (0.76 ~ 1.43)	0.792		
ECOG PS				
0	1.00 (Reference)			
1	1.07 (0.78 ~ 1.46)	0.673		
AFP (ng/mL)				
≤400	1.00 (Reference)		1.00 (Reference)	
>400	1.53 (1.11 ~ 2.10)	0.008	0.76 (0.49 ~ 1.17)	0.212
Tumor diameter(cm)				
5.1-10	1.00 (Reference)		1.00 (Reference)	
>10	3.86 (2.62 ~ 5.70)	<0.001	6.03(3.56 ~ 10.20)	<0.001
Tumor number				
≤3	1.00 (Reference)		1.00 (Reference)	
>3	1.37 (0.97 ~ 1.93)	0.070	1.33 (0.93 ~ 1.90)	0.113
PVTT grade				
Vp1-2	1.00 (Reference)		1.00 (Reference)	
Vp3-4	1.59 (1.09 ~ 2.33)	0.017	2.01 (1.36 ~ 2.97)	<.0001
Extrahepatic metastasis				
No	1.00 (Reference)			
Yes	1.14 (0.67 ~ 1.94)	0.633		

PSM, Propensity Score Matching; HR, Hazard Ratio; CI, Confidence Interval; TAC, TACE combined with apatinib plus camrelizumab therapy; THAC, TACE-HAIC combined with apatinib plus camrelizumab therapy; HBV, Hepatitis B Virus; ECOG PS, Eastern Cooperative Oncology Group Performance Status; AFP, Alpha-fetoprotein; PVTT, Portal vein tumor thrombosis.

**Table 4:** Predictors of overall survival after PSM.

Variables	Univariable analysis		Multivariable analysis	
	HR (95%CI)	*P*	HR (95%CI)	*P*
Treatment Method				
TAC	1.00 (Reference)		1.00 (Reference)	
THAC	0.20 (0.14 ~ 0.29)	<0.001	0.13 (0.08 ~ 0.19)	<0.001
Gender				
Male	1.00 (Reference)			
Female	1.07 (0.52 ~ 2.20)	0.853		
Age(y)				
≤60	1.00 (Reference)			
>60	0.96 (0.69 ~ 1.34)	0.813		
HBV infection				
No	1.00 (Reference)		1.00 (Reference)	
Yes	0.66 (0.41 ~ 1.04)	0.073	0.75 (0.45 ~ 1.25)	0.268
Cirrhosis				
No	1.00 (Reference)			
Yes	1.29 (0.83 ~ 2.00)	0.260		
Child-Pugh class				
A	1.00 (Reference)			
B	0.91 (0.65 ~ 1.26)	0.564		
ECOG PS				
0	1.00 (Reference)			
1	0.92 (0.66 ~ 1.28)	0.619		
AFP (ng/mL)				
≤400	1.00 (Reference)			
>400	1.21 (0.87 ~ 1.68)	0.263		
Tumor diameter(cm)				
5.1-10	1.00 (Reference)		1.00 (Reference)	
>10	2.66 (1.83 ~ 3.88)	<0.001	4.01 (2.70 ~ 5.96)	<0.001
Tumor number				
≤3	1.00 (Reference)		1.00 (Reference)	
>3	1.36 (0.95 ~ 1.94)	0.097	1.49 (1.01 ~ 2.22)	0.046
PVTT grade				
Vp1-2	1.00 (Reference)		1.00 (Reference)	
Vp3-4	1.75 (1.17 ~ 2.63)	0.007	1.82 (1.19 ~ 2.78)	0.006
Extrahepatic metastasis				
No	1.00 (Reference)		1.00 (Reference)	
Yes	3.26 (1.87 ~ 5.71)	<0.001	4.78 (2.65 ~ 8.62)	<0.001

PSM, Propensity Score Matching; HR, Hazard Ratio; CI, Confidence Interval; TAC, TACE combined with apatinib plus camrelizumab therapy; THAC, TACE-HAIC combined with apatinib plus camrelizumab therapy; HBV, Hepatitis B Virus. ECOG PS, Eastern Cooperative Oncology Group Performance Status; AFP, Alpha-fetoprotein; PVTT, Portal vein tumor thrombosis.

### Subgroup analysis

After PSM, prespecified subgroup analyses were performed for PFS (**Figure 5A**) and OS (**Figure 5B**). Compared with the TAC regimen, the THAC regimen was associated with superior PFS across all predefined subgroups. In addition, THAC demonstrated improved OS in most of the analyzed subgroups. However, no statistically significant OS benefit was observed among female patients or patients without HBV infection. This finding may be attributable to the limited sample size within these subgroups. Detailed results of the subgroup analyses are presented in **Figure 5**.

**Figure 5:**
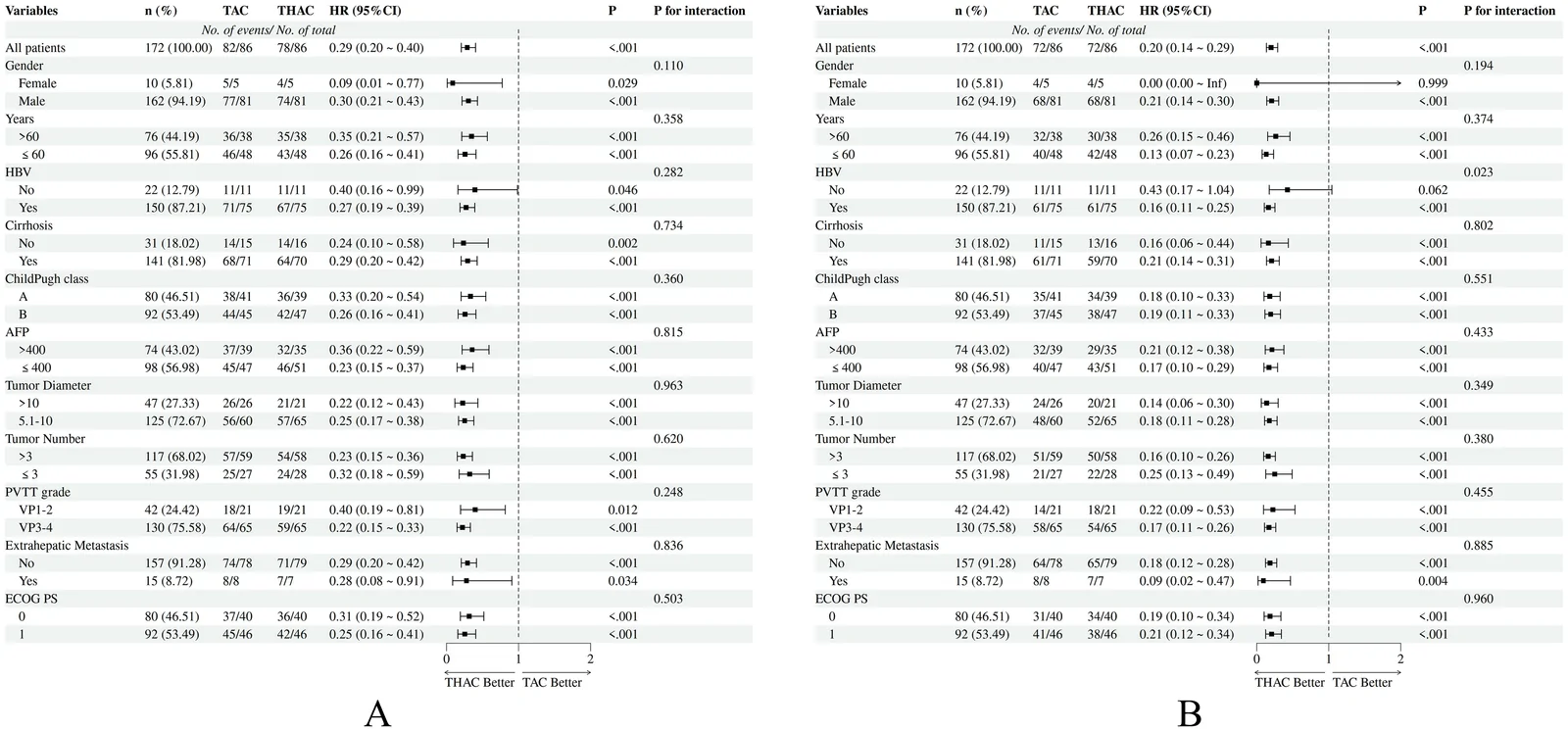
Forest plot of subgroup analysis for PFS **(A)** and OS **(B)**.

### Adverse events

After PSM, adverse events were compared between the two groups (**Table 5**). No significant differences were observed in the incidence of adverse events of any grade between the THAC and TAC groups (all P>0.05). Similarly, the incidence of grade 3 or higher adverse events did not differ significantly between the two groups (all P>0.05). Notably, no grade 5 adverse events were observed in the entire cohort. In the THAC group, 8 patients (9.3%) required dose reduction or temporary interruption of apatinib because of adverse events, and 2 patients (2.3%) required temporary interruption of camrelizumab. In the TAC group, 6 patients (7.0%) required dose reduction or temporary interruption of apatinib, 1 patient (1.2%) required temporary interruption of camrelizumab, and 1 patient (1.2%) permanently discontinued camrelizumab. No treatment-related deaths occurred during the study period.

**Table 5:** Comparison of treatment-related adverse events between the two groups after PSM.

Adverse events	All grade			Grade≥3		
	THAC group (n = 86)	TAC group (n = 86)	P value	THAC group (n = 86)	TAC group (n = 86)	P value
Fever	62(72.1%)	58(67.4%)	0.507	0	0	-
Abdominal pain	57(66.3%)	64(74.4%)	0.167	0	0	-
Nausea	59(68.6%)	53(61.6%)	0.337	0	0	-
Vomiting	48(55.8%)	39(45.3%)	0.170	4(4.6%)	2(2.3%)	0.678
Diarrhea	35(40.7%)	38(44.2%)	0.643	2(2.3%)	3(3.5%)	1.000
Hypertension	34(39.5%)	31(36.0%)	0.637	2(2.3%)	1(1.2%)	1.000
Hand-foot skin reaction	36(41.9%)	32(37.2%)	0.533	6(7.0%)	4(4.6%)	0.745
Fatigue	30(34.9%)	28(32.6%)	0.747	0	0	-
Weight loss	37(43.0%)	41(47.7%)	0.540	0	0	-
Anorexia	44(51.2%)	49(57.0%)	0.444	5(5.8%)	4(4.6%)	1.000
Hypothyroidism	13(15.1%)	15(17.4%)	0.680	0	0	-
Rash	37(43.0%)	32(37.2%)	0.437	1(1.2%)	1(1.2%)	1.000
Proteinuria	33(38.4%)	34(39.5%)	0.876	1(1.2%)	2(2.3%)	1.000
Gastrointestinal bleeding	6(7.0%)	4(4.7%)	0.745	0	0	-
Elevated bilirubin	40(46.5%)	44(51.2%)	0.542	5(5.8%)	8(9.3%)	0.387
Thrombocytopenia	49(57%)	45(52.3%)	0.540	11(12.8%)	10(11.6%)	0.971
Leukopenia	46(53.5%)	41(47.7%)	0.446	9(10.5%)	7(8.1%)	0.600
Elevated AST	58(67.4%)	63(73.3%)	0.404	14(16.3%)	17(19.8%)	0.552
Elevated ALT	64(74.4%)	67(77.9%)	0.591	16(18.6%)	21(24.4%)	0.195
Gingival bleeding	12(14.0%)	9(10.5%)	0.485	0	0	-
Oral mucositis	18(20.9%)	13(15.1%)	0.321	0	0	-
RCCEP	29(33.7%)	21(24.4%)	0.179	2(2.3%)	1(1.2%)	1.000
Immune-related dermatitis	4(4.6%)	3(3.5%)	1.000	0	0	-
Immune-related pneumonitis	3(3.5%)	2(2.3%)	1.000	0	0	-
Immune-related hepatitis	5(5.8%)	7(8.1%)	0.549	0	1(1.2%)	1.000
Immune-related myocarditis	1(1.2%)	2(2.3%)	1.000	0	0	-

PSM, Propensity Score Matching; HR, Hazard Ratio; CI, Confidence Interval; TAC, TACE combined with apatinib plus camrelizumab therapy; THAC, TACE-HAIC combined with apatinib plus camrelizumab therapy; AST, Aspartate aminotransferase; ALT, Alanine aminotransferase; RCCEP, reactive cutaneous capillary endothelial proliferation.

## Discussion

In this multicenter retrospective study, we compared the efficacy and safety of the THAC regimen and the TAC regimen in patients with unresectable hepatocellular carcinoma (uHCC) accompanied by portal vein tumor thrombus (PVTT). The results demonstrated that, compared with the TAC regimen, the THAC regimen significantly improved the ORR and DCR of both intrahepatic tumors and PVTT, and was associated with prolonged PFS and OS. Notably, these advantages remained consistent after PSM, further supporting the robustness of our findings. Multivariable analysis identified tumor diameter and PVTT classification as independent risk factors for PFS, whereas tumor diameter, tumor number, PVTT classification, and extrahepatic metastasis were independent risk factors for OS. In addition, the THAC regimen was identified as an independent protective factor for both PFS and OS. Furthermore, subgroup analyses demonstrated that the THAC regimen provided superior survival benefits across most patient subgroups, with a particularly pronounced benefit observed among patients with a high tumor burden. In terms of safety, no significant differences were observed between the two groups in the overall incidence of adverse events of any grade or grade 3 or higher adverse events. Most treatment-related adverse events were mild and manageable.

The superior efficacy of the THAC regimen compared with the TAC regimen may be attributable to the synergistic antitumor effects of TACE, HAIC, apatinib, and camrelizumab. HAIC enables the continuous delivery of high concentrations of chemotherapeutic agents directly into the hepatic artery, thereby increasing local drug exposure within the tumor while reducing systemic drug concentrations and minimizing treatment-related toxicity. In addition, HAIC may compensate for the limitations of TACE, which can result in incomplete embolization because of occult tumor-feeding arteries that are difficult to identify during the procedure, thereby enhancing overall antitumor efficacy (27). A study by Liu et al. (28) demonstrated that, in patients with unresectable massive hepatocellular carcinoma, TACE combined with HAIC significantly improved ORR and DCR and prolonged both PFS and OS compared with TACE alone. In addition, chemotherapeutic agents administered during HAIC may improve the tumor immune microenvironment by enhancing the infiltration of CD8^+^ T cells into tumor tissues. Meanwhile, camrelizumab restores antitumor immunity through blockade of the PD-1/PD-L1 pathway, thereby promoting T-cell–mediated immune responses. Therefore, HAIC may enhance the therapeutic efficacy of camrelizumab, and the combination of the two treatments may exert synergistic antitumor effects, contributing to improved clinical outcomes (10, 29). TACE may induce local hypoxia within tumor tissues, leading to upregulation of vascular endothelial growth factor (VEGF) expression and consequently increasing the risk of tumor progression and metastasis (30). Apatinib, a selective VEGFR-2 inhibitor, can counteract this effect by suppressing VEGFR-2–mediated angiogenic signaling and promoting tumor vascular normalization, thereby inhibiting tumor recurrence and progression (31). Li et al. (32) reported that, compared with TACE alone, TACE combined with apatinib improved ORR and prolonged PFS in patients with uHCC. However, TACE-induced upregulation of VEGF not only promotes tumor angiogenesis but may also disrupt the interaction between leukocytes and endothelial cells, thereby impairing immune-cell infiltration into the tumor microenvironment (33). Therefore, beyond its antiangiogenic activity, blockade of the VEGF pathway by apatinib may facilitate the infiltration of effector T cells into tumor tissues, thereby enhancing the antitumor immune response mediated by camrelizumab. Duan et al. (34) further demonstrated that the addition of camrelizumab to TACE plus apatinib was associated with improved survival outcomes in patients with uHCC. Furthermore, the multicenter randomized phase II CHANCE-005/CARES-005 trial similarly demonstrated that TACE combined with apatinib and camrelizumab significantly prolonged PFS in patients with uHCC. Collectively, these findings indicate that adding the systemic regimen of apatinib plus camrelizumab to TACE yields superior survival benefits for patients with uHCC (35).Moreover, several studies have shown that TACE-HAIC combined with targeted therapy and immunotherapy can significantly improve tumor control and prolong survival in patients with HCC (29, 36, 37). These findings provide clinical support for the integration of locoregional and systemic therapies and may help explain the superior efficacy of the THAC regimen observed in the present study.

Taken together, the superior efficacy of the THAC regimen may be attributed to the synergistic effects of locoregional therapy, systemic therapy, and immune modulation. TACE can rapidly reduce tumor burden, while HAIC further increases local exposure to chemotherapeutic agents and provides sustained control of residual tumor lesions. Apatinib inhibits tumor angiogenesis and promotes vascular normalization, thereby creating a more favorable environment for immune-cell infiltration. Meanwhile, camrelizumab restores T cell–mediated antitumor immunity and enhances immune surveillance. These therapeutic modalities may act synergistically at multiple levels to facilitate tumor cell eradication, vascular remodeling, and improvement of the tumor immune microenvironment, ultimately contributing to improved tumor control and survival outcomes.

Notably, all patients included in the present study had concomitant PVTT. PVTT is a hallmark of the highly invasive nature of HCC and is also an important contributor to poor prognosis. Our results demonstrated that the THAC regimen not only significantly improved the response of intrahepatic primary tumors but also achieved superior therapeutic responses in PVTT. Patients in the THAC group exhibited better treatment responses in both intrahepatic lesions and PVTT, accompanied by significantly prolonged PFS and OS. These findings suggest that the THAC regimen has considerable therapeutic potential for patients with uHCC and PVTT. Consistent with our findings, Yuan et al. (38) reported that TACE-HAIC combined with targeted therapy and immunotherapy achieved favorable clinical outcomes in patients with HCC and PVTT.

Despite the encouraging findings, several limitations of this study should be acknowledged. First, as a multicenter retrospective study, selection bias could not be completely avoided. Although propensity score matching was performed to balance baseline characteristics between the two groups, residual confounding resulting from unmeasured variables may still have been present. Second, the sample size after matching was relatively limited, which may have reduced the statistical power of certain subgroup analyses and affected the precision of treatment-effect estimates. Therefore, these findings require further validation in larger cohorts. Third, detailed treatment-exposure data were not systematically recorded for each patient, including the number of TACE/HAIC sessions, the actual duration of apatinib therapy, and the number of camrelizumab cycles. Therefore, differences in actual treatment exposure between the two groups could not be fully evaluated, which may have affected the interpretation of the observed survival differences. Finally, the follow-up duration was relatively short and may not have been sufficient to fully assess the long-term survival benefits and cumulative treatment-related toxicities associated with this combination regimen. Future prospective randomized controlled trials with larger sample sizes and longer follow-up are warranted to validate our findings and further define the clinical value of this intensified multimodal treatment strategy in patients with uHCC and PVTT.

## Conclusion

In conclusion, the combination of TACE-HAIC, apatinib, and camrelizumab demonstrated promising antitumor activity in patients with uHCC and PVTT, providing effective control of both intrahepatic tumors and PVTT while significantly improving survival outcomes. With an acceptable safety profile and no apparent increase in severe toxicity, this regimen represents a promising therapeutic option for this patient population.

## Data Availability

The raw data supporting the conclusions of this article will be made available by the authors, without undue reservation.

## References

[1] Liang Y Yang J Li H Wu J Tan W Ren Y . Efficacy and safety of TACE plus lenvatinib with PD-1 inhibitor for unresectable huge hepatocellular carcinoma: a multicenter retrospective cohort study. Int J Surg. (2025). doi: 10.1097/JS9.0000000000004131 41347264

[2] Huang J Huang W Zhan M Guo Y Liang L Cai M . Drug-eluting bead transarterial chemoembolization combined with FOLFOX-based hepatic arterial infusion chemotherapy for large or huge hepatocellular carcinoma. J Hepatocell Carcinoma. (2021) 8:1445–58. doi: 10.2147/JHC.S339379 34858889 PMC8631985

[3] Liu X Yin L Liu B Hou X Li Y Liu R . TACE-HAIC plus targeted therapy and immunotherapy for HCC with tumor thrombus and arteriovenous fistula. J Hepatocell Carcinoma. (2025) 12:2951–69. doi: 10.2147/JHC.S567414 41467098 PMC12744873

[4] Li X Ding X Liu M Wang J Sun W Teng Y . A multicenter prospective study of TACE combined with lenvatinib and camrelizumab for hepatocellular carcinoma with portal vein tumor thrombus. Cancer Med. (2023) 12:16805–14. doi: 10.1002/cam4.6302 37387602 PMC10501288

[5] Cai M Liang L Zhang J Chen N Huang W Guo Y . Lenvatinib plus drug-eluting bead transarterial chemoembolization with/without hepatic arterial infusion chemotherapy for hepatocellular carcinoma larger than 7 cm with major portal vein tumor thrombosis: a multicenter retrospective cohort study. Int J Surg. (2024) 110:7860–70. doi: 10.1097/JS9.0000000000001819 38869974 PMC11634077

[6] Marrero JA Kulik LM Sirlin CB Zhu AX Finn RS Abecassis MM . Diagnosis, staging, and management of hepatocellular carcinoma: 2018 practice guidance by the American Association for the Study of Liver Diseases. Hepatology. (2018) 68:723–50. doi: 10.1002/hep.29913 29624699

[7] European Association for the Study of the L . EASL clinical practice guidelines on hepatitis delta virus. J Hepatol. (2023) 79:433–60. doi: 10.1016/j.jhep.2023.05.001 37364791

[8] Xiang X Lau WY Wu ZY Zhao C Ma YL Xiang BD . Transarterial chemoembolization versus best supportive care for patients with hepatocellular carcinoma with portal vein tumor thrombus: a multicenter study. Eur J Surg Oncol. (2019) 45:1460–7. doi: 10.1016/j.ejso.2019.03.042 31005471

[9] Sun J Guo R Bi X Wu M Tang Z Lau WY . Guidelines for diagnosis and treatment of hepatocellular carcinoma with portal vein tumor thrombus in China (2021 edition). Liver Cancer. (2022) 11:315–28. doi: 10.1159/000523997 35978596 PMC9294940

[10] Wu S Qiu F Zhang F Chen P Zheng X Wei Q . Hepatic arterial infusion chemotherapy combined with apatinib plus camrelizumab for advanced hepatocellular carcinoma with type Vp4 portal vein tumor thrombosis: a multicenter propensity score-matching analysis. Front Immunol. (2026) 17:1742116. doi: 10.3389/fimmu.2026.1742116 41782861 PMC12953477

[11] Zeng D Cheng Z Lu J Liu G Li B . Efficacy of hepatic arterial infusion chemotherapy in advanced hepatocellular carcinoma: survival outcomes and prognostic factors from a systematic review and meta-analysis. Hepatobiliary Surg Nutr. (2025) 14:587–607. doi: 10.21037/hbsn-2025-115 40893755 PMC12393126

[12] Liu M Shi J Mou T Wang Y Wu Z Shen A . Systematic review of hepatic arterial infusion chemotherapy versus sorafenib in patients with hepatocellular carcinoma with portal vein tumor thrombosis. J Gastroenterol Hepatol. (2020) 35:1277–87. doi: 10.1111/jgh.15010 32052876

[13] Leung JH Wang SY Leung HWC Chan ALF . Comparative efficacy and safety of multimodality treatment for advanced hepatocellular carcinoma with portal vein tumor thrombus: patient-level network meta-analysis. Front Oncol. (2024) 14:1344798. doi: 10.3389/fonc.2024.1344798 38434681 PMC10905023

[14] Kudo M Kawamura Y Hasegawa K Tateishi R Kariyama K Shiina S . Management of hepatocellular carcinoma in Japan: JSH consensus statements and recommendations 2021 update. Liver Cancer. (2021) 10:181–223. doi: 10.1159/000514174 34239808 PMC8237791

[15] Chen S Wang X Yuan B Peng J Zhang Q Yu W . Apatinib plus hepatic arterial infusion of oxaliplatin and raltitrexed for hepatocellular carcinoma with extrahepatic metastasis: phase II trial. Nat Commun. (2024) 15:8857. doi: 10.1038/s41467-024-52700-z 39402023 PMC11473759

[16] Qin S Li Q Gu S Chen X Lin L Wang Z . Apatinib as second-line or later therapy in patients with advanced hepatocellular carcinoma (AHELP): a multicentre, double-blind, randomised, placebo-controlled, phase 3 trial. Lancet Gastroenterol Hepatol. (2021) 6:559–68. doi: 10.1016/S2468-1253(21)00109-6 33971141

[17] Xia Y Tang W Qian X Li X Cheng F Wang K . Efficacy and safety of camrelizumab plus apatinib during the perioperative period in resectable hepatocellular carcinoma: a single-arm, open label, phase II clinical trial. J Immunother Cancer. (2022) 10(4):e004656. doi: 10.1136/jitc-2022-004656 35379737 PMC8981365

[18] Finn RS Qin S Ikeda M Galle PR Ducreux M Kim TY . Atezolizumab plus bevacizumab in unresectable hepatocellular carcinoma. N Engl J Med. (2020) 382:1894–905. doi: 10.1056/NEJMoa1915745 32402160

[19] Yuan G Cheng X Li Q Zang M Huang W Fan W . Safety and efficacy of camrelizumab combined with apatinib for advanced hepatocellular carcinoma with portal vein tumor thrombus: a multicenter retrospective study. Onco Targets Ther. (2020) 13:12683–93. doi: 10.2147/OTT.S286169 33328740 PMC7734076

[20] Xu J Shen J Gu S Zhang Y Wu L Wu J . Camrelizumab in combination with apatinib in patients with advanced hepatocellular carcinoma (RESCUE): a nonrandomized, open-label, phase II trial. Clin Cancer Res. (2021) 27:1003–11. doi: 10.1158/1078-0432.CCR-20-2571 33087333

[21] Qin S Gu S Chan SL Bai Y Ren Z Lin X . Camrelizumab plus rivoceranib versus sorafenib as first-line therapy for unresectable hepatocellular carcinoma (CARES-310): final analysis of a randomised, open-label, international, phase 3 study. Lancet Oncol. (2025) 26:1598–611. doi: 10.1016/S1470-2045(25)00543-1 41308676

[22] Zhang TQ Geng ZJ Zuo MX Li JB Huang JH Huang ZL . Camrelizumab (a PD-1 inhibitor) plus apatinib (an VEGFR-2 inhibitor) and hepatic artery infusion chemotherapy for hepatocellular carcinoma in Barcelona Clinic Liver Cancer stage C (TRIPLET): a phase II study. Signal Transduct Target Ther. (2023) 8:413. doi: 10.1038/s41392-023-01663-6 37884523 PMC10603153

[23] Lu H Zheng C Liang B Xia X Fan H . Efficacy and safety analysis of TACE + PEI + lenvatinib compared with TACE + lenvatinib for the treatment of hepatocellular carcinoma with PVTT: a retrospective study. Front Oncol. (2024) 14:1280837. doi: 10.3389/fonc.2024.1280837 38298738 PMC10827889

[24] Yin L Li W Shao W Hou X Liu B Liu X . TACE-HAlC combined with Donafenib and immune checkpoint inhibitors for BCLC stage C HCC patients (THEME study): a retrospective IPTW-adjusted cohort study. Front Immunol. (2025) 16:1669856. doi: 10.3389/fimmu.2025.1669856 41357217 PMC12676704

[25] Freites-Martinez A Santana N Arias-Santiago S Viera A . Using the Common Terminology Criteria for Adverse Events (CTCAE - Version 5.0) to evaluate the severity of adverse events of anticancer therapies. Actas Dermosifiliogr (Engl Ed). (2021) 112:90–2. doi: 10.1016/j.ad.2019.05.009 32891586

[26] Lencioni R Llovet JM . Modified RECIST (mRECIST) assessment for hepatocellular carcinoma. Semin Liver Dis. (2010) 30:52–60. doi: 10.1055/s-0030-1247132 20175033 PMC12268942

[27] Ma Y Long J Shi S Fang X Wang R Xiong F . DEB-TACE-HAIC combined with donafenib and camrelizumab in the treatment of unresectable hepatocellular carcinoma: a multicenter retrospective study. Front Immunol. (2026) 17:1779170. doi: 10.3389/fimmu.2026.1779170 42292418 PMC13260537

[28] Liu C Li J Zhao M Zheng L Xiao JC . TACE-HAIC versus TACE as initial regional therapy for unresectable huge hepatocellular carcinoma (>10 cm): a propensity score-matched study. J Hepatocell Carcinoma. (2025) 12:2083–93. doi: 10.2147/JHC.S537950 40980779 PMC12448089

[29] Liu B Yin L Chen Y Hou X Li Y Liu X . Efficacy and safety of TACE-HAIC combined with tyrosine kinase inhibitors and immune checkpoint inhibitors for the patients with BCLC-defined stage B-C HCC. Front Oncol. (2025) 15:1615506. doi: 10.3389/fonc.2025.1615506 40799236 PMC12340405

[30] Huang Z Chen T Li W He W Liu S Wu Z . Atezolizumab and bevacizumab plus transarterial chemoembolization and hepatic arterial infusion chemotherapy for patients with high tumor burden unresectable hepatocellular carcinoma: a multi-center cohort study. Int Immunopharmacol. (2024) 139:112711. doi: 10.1016/j.intimp.2024.112711 39029233

[31] Tian Z Niu X Yao W . Efficacy and response biomarkers of apatinib in the treatment of malignancies in China: a review. Front Oncol. (2021) 11:749083. doi: 10.3389/fonc.2021.749083 34676173 PMC8525889

[32] Li N Yang P Fang J . Transarterial chemoembolization (TACE) plus apatinib vs. TACE alone for hepatocellular carcinoma. Clin Res Hepatol Gastroenterol. (2022) 46:102022. doi: 10.1016/j.clinre.2022.102022 36089248

[33] Cheu JW Wong CC . Mechanistic rationales guiding combination hepatocellular carcinoma therapies involving immune checkpoint inhibitors. Hepatology. (2021) 74:2264–76. doi: 10.1002/hep.31840 33811765

[34] Duan X Li H Kuang D Chen P Zhang K Li Y . Transcatheter arterial chemoembolization plus apatinib with or without camrelizumab for unresectable hepatocellular carcinoma: a multicenter retrospective cohort study. Hepatol Int. (2023) 17:915–26. doi: 10.1007/s12072-023-10519-8 37012542 PMC10386927

[35] Zhu HD Fan WJ Zhao C Wang S Li YL Jin ZC . Transarterial chemoembolization combined with camrelizumab and rivoceranib for unresectable hepatocellular carcinoma (CHANCE2005/CARES-005): a randomized phase II trial. J Clin Oncol. (2026) 44:959–69. doi: 10.1200/JCO-25-01796 41734362 PMC13075789

[36] Wu C Dong Y Li X Shao W Wang G Wu H . TACE-HAIC versus HAIC combined with TKIs and ICIs for hepatocellular carcinoma with a high tumor burden-a propensity-score matching comparative study. Front Immunol. (2025) 16:1664756. doi: 10.3389/fimmu.2025.1664756 41368633 PMC12682802

[37] Pang B Zuo B Huang L You X Liu T Hao J . Real-world efficacy and safety of TACE-HAIC combined with TKIs and PD-1 inhibitors in initially unresectable hepatocellular carcinoma. Int Immunopharmacol. (2024) 137:112492. doi: 10.1016/j.intimp.2024.112492 38906005

[38] Yuan Y He W Yang Z Qiu J Huang Z Shi Y . TACE-HAIC combined with targeted therapy and immunotherapy versus TACE alone for hepatocellular carcinoma with portal vein tumour thrombus: a propensity score matching study. Int J Surg. (2023) 109:1222–30. doi: 10.1097/JS9.0000000000000256 37026861 PMC10389515

